# Comparison of base-line and chemical-induced transcriptomic responses in HepaRG and RPTEC/TERT1 cells using TempO-Seq

**DOI:** 10.1007/s00204-018-2256-2

**Published:** 2018-07-14

**Authors:** Alice Limonciel, Gamze Ates, Giada Carta, Anja Wilmes, Manfred Watzele, Peter J. Shepard, Harper C. VanSteenhouse, Bruce Seligmann, Joanne M. Yeakley, Bob van de Water, Mathieu Vinken, Paul Jennings

**Affiliations:** 1 Division of Molecular and Computational Toxicology, Amsterdam Institute for Molecules, Medicines and Systems, Vrije Universiteit Amsterdam, De Boelelaan 1108, 1081 HZ Amsterdam, The Netherlands; 2 Department of In Vitro Toxicology and Dermato-Cosmetology, Vrije Universiteit Brussel, Laarbeeklaan 103, 1090 Brussels, Belgium; 3 Division of Physiology, Department of Physiology and Medical Physics, Medical University of Innsbruck, 6020 Innsbruck, Austria; 4 Roche Diagnostics GmbH, Nonnenwald 2, 82377 Penzberg, Germany; 5 BioSpyder Technologies, Inc., 5922 Farnsworth Ct Ste 102, Carlsbad, CA 92008 USA; 6 Division of Toxicology, Leiden Academic Center for Drug Research, Leiden University, PO Box 9503, 2300 RA Leiden, The Netherlands

**Keywords:** HepaRG, RPTEC/TERT1, TempO-Seq, Stress responses, Dedifferentiation

## Abstract

The utilisation of genome-wide transcriptomics has played a pivotal role in advancing the field of toxicology, allowing the mapping of transcriptional signatures to chemical exposures. These activities have uncovered several transcriptionally regulated pathways that can be utilised for assessing the perturbation impact of a chemical and also the identification of toxic mode of action. However, current transcriptomic platforms are not very amenable to high-throughput workflows due to, high cost, complexities in sample preparation and relatively complex bioinformatic analysis. Thus, transcriptomic investigations are usually limited in dose and time dimensions and are, therefore, not optimal for implementation in risk assessment workflows. In this study, we investigated a new cost-effective, transcriptomic assay, TempO-Seq, which alleviates the aforementioned limitations. This technique was evaluated in a 6-compound screen, utilising differentiated kidney (RPTEC/TERT1) and liver (HepaRG) cells and compared to non-transcriptomic label-free sensitive endpoints of chemical-induced disturbances, namely phase contrast morphology, xCELLigence and glycolysis. Non-proliferating cell monolayers were exposed to six sub-lethal concentrations of each compound for 24 h. The results show that utilising a 2839 gene panel, it is possible to discriminate basal tissue-specific signatures, generate dose–response relationships and to discriminate compound-specific and cell type-specific responses. This study also reiterates previous findings that chemical-induced transcriptomic alterations occur prior to cytotoxicity and that transcriptomics provides in depth mechanistic information of the effects of chemicals on cellular transcriptional responses. TempO-Seq is a robust transcriptomic platform that is well suited for in vitro toxicity experiments.

**Electronic supplementary material:**

The online version of this article (10.1007/s00204-018-2256-2) contains supplementary material, which is available to authorized users.

## Introduction

A distinct advantage of in vitro techniques over whole animal models is their applicability to mechanistic investigations and the ability to use cells of human origin. Mechanistic-based assays are rapidly becoming the corner-stone of contemporary toxicological investigations, driven by advances in genetic analysis and associated omic methodologies. Transcriptomics has been a key tool in allowing a better understanding of how the cellular program is altered in response to stress situations (Jennings et al. 2013). However, whole genome arrays are still too expensive to be considered for routine use. Additionally, RNA sample preparation and post-analysis is cumbersome when sample numbers are large. Thus, multiple compound concentrations and/or temporal effects are seldom studied in transcriptomic investigations and toxicogenomic data is often limited in dimension (Wilmes et al. 2011, 2013). Where complete dose responses were conducted, for example, by Waldmann et al., the chemical-induced alterations in transcriptomic expression were shown to manifest at concentrations well below alterations in cell viability (Waldmann et al. 2014). Thus, if transcriptomic assays become cheaper and easier to perform, it is likely to become a dominant methodology for hazard and risk assessment, due to the wealth of mechanistic information it provides.

In the present study we investigated the utility of a new, cost-effective technique templated oligo assay with sequencing readout (TempO-Seq) that solves both the cost and throughput issues (Grimm et al. 2016; Yeakley et al. 2017). TempO-Seq is an NGS library preparation method that is based on ligation of detector oligos that are hybridized directly to RNA targets, with the subsequent addition of sample barcodes and sequencing adapters by PCR amplification. In addition, there is no RNA extraction step required. The detector oligos are chimeric and contain a sequence specific to the targeted RNA as well as a sequence in common among the detector oligos for universal primers. TempO-Seq thus allows cost-effective, simultaneous quantification of specific mRNA targets without the need to isolate RNA. In this particular study, we utilised a probe set identifying 2839 genes which is a combination of a previously selected gene panel (Mav et al. 2018) supplemented with knowledge-based cellular stress response-related genes identified by the experiences of the academic groups in this publication. The entire gene set utilised is provided in Table S1. The Mav et al. gene-set was developed by the U.S. Tox21 Federal collaboration program and represents a data-driven strategy of sentinel genes (selected for biological diversity, maximal information content, and widespread pathway coverage), which was also augmented using a knowledge-driven selection of additional genes (Mav et al. 2018).

Two of the major target organs for systemic toxicology are the liver and kidney. While renal and hepatic in vitro systems are often run within the same project umbrella, they are not usually challenged with the same compounds at the same concentrations. This makes it difficult to compare data and draw conclusions pertaining to tissue-specificity of responses and tissue-specific biomarkers. Here we utilised a human renal and human hepatic cell line (RPTEC/TERT1 and HepaRG, respectively). RPTEC/TERT1 and HepaRG are considered the most differentiated and stable cell lines currently available for their respective tissues and thus the most applicable for in vitro toxicological investigations (Guillouzo et al. 2007; Doktorova et al. 2013; Aschauer et al. 2013, 2015). Both cell types were used for drug exposures under serum-free conditions and under differentiated non-proliferating monolayer conditions. The cells were exposed to the same six compounds at the same concentrations, measuring the same endpoints (impedance, glycolysis, morphology, and targeted TempO-Seq transcriptomics). The six structurally unrelated compounds selected were ochratoxin A (OTA), potassium bromate (KBrO_3_), cyclosporine A (CsA), acetaminophen (APAP), isoniazid (ISZ) and sodium valproate (VALP). While some of these may be considered preferentially nephrotoxic or hepatotoxic, in reality, many adversely affect both organs and in addition pharmacokinetic properties are likely to play a major role in target organ specificity in vivo.

The present study demonstrates the usefulness of the TempO-Seq methodology, identifies differences in RPTEC/TERT1 and HepaRG expressed transcriptomes, differences in toxicological responses and identifies chemical- and tissue-specificity of certain gene and pathway responses.

## Materials and methods

### Routine cell culture and differentiation

The hepatic cell line, HepaRG (Guillouzo et al. 2007), was obtained from BioPredic International and the renal proximal tubule cell line, RPTEC/TERT1 (Wieser et al. 2008), from Evercyte GmbH. HepaRG cells were routinely cultured in William’s E medium (Gibco 12551032) supplemented with 2 mM Glutamax, 5 µg/ml insulin, 50 µM hydrocortisone 21-hemisuccinate, 100 U/ml penicillin, 100 µg/ml streptomycin and 9% Foetal Calf Serum (FCS) (all components were from BioPredic International). RPTEC/TERT1 cells were routinely cultured, differentiated and exposed to chemicals in a 1:1 mix of DMEM (Gibco 11966-025) and Ham’s F12 (Gibco 21765-029) (containing a final concentration of 5 mM glucose) and supplemented with 2 mM Glutamax, 10 ng/ml epidermal growth factor, 36 ng/ml hydrocortisone, 5 µg/ml insulin, 5 µg/ml transferrin, 5 ng/ml selenium, 100 U/ml penicillin and 100 µg/ml streptomycin (Jennings et al. 2009). Cells were cultured in a controlled humidified 37 °C, 5% CO_2_ environment. Cells were routinely passaged once a week in trypsin EDTA. HepaRG cells were differentiated according to Biopredic’s 6 day protocol (Biopredic International 2017). Briefly, cells were seeded on 10 µg/cm^2^ collagen I (Biopredic) coated 12 well or 96 well plates in base William’s E medium with GlutaMAX and ADD670 additives (Biopredic). Medium was changed after 24 h to maintenance/metabolism medium (base medium with ADD620 additives) and renewed after 48 h. Three days later the medium was changed to Induction medium (base medium with ADD650 serum-free additives). RPTEC/TERT1 cells were differentiated by allowing them to reach confluence and remain in a confluent state for at least 7 days before treatment as previously described (Aschauer et al. 2013).

### Chemical exposures

From the past experiences of the two experienced testing laboratories (i.e., Medical University of Innsbruck, MUI, and the Vrije Universiteit Brussel, VUB), a panel of six unrelated compounds and associated concentrations were selected (Table 1). Differentiated cell monolayers were washed and treated with the compounds in serum-free medium for 24 h. All compounds, except for CsA, were water soluble. Stock solutions of CsA (15 mM) were made in 100% DMSO, aliquoted and frozen. All final concentrations for CsA had a 0.1% DMSO content and 0.1% DMSO was used as a vehicle control. OTA was made up to 2.48 mM in supplemented DMEM/Ham’s F12 aliquoted and frozen. All other stocks were generated in either HepaRG complete medium or RPTEC/TERT1 complete medium and used freshly (Table 1).

**Table 1:** Concentrations of chemicals used with vehicle and ordering information

	Tested concentrations in µM					
Name	Ochratoxin A	Potassium Bromate	Cyclosporine A	Na valproate	Acetaminophen	Isoniazid
Short name	OTA	KBrO_3_	CsA	VALP	APAP	ISZ
Dilution						
0	0	0	0	0	0	0
1	0.001	80	0.05	8	8	8
2	0.01	400	1	40	40	40
3	0.13	800	5	200	200	200
4	1	2000	10	1000	1000	1000
5	10	4000	15	5000	5000	10,000
Pre-stock	2.48 mM	–	15 mM	–	–	–
Vehicle	RPTEC/TERT1 Medium	RPTEC/TERT1 Medium	DMSO	HepaRG medium	HepaRG medium	HepaRG medium
Source	Sigma	Sigma	Calbiochem	Sigma	Sigma	Sigma
Cat No	O1877	P7332	239835	P4543	A7302	I3377

0–5 are the dilutions, all values are expressed in µM unless otherwise stated

### xCELLigence assay

xCELLigence experiments were conducted at the MUI laboratory. Cells were seeded in the proprietary E-Plates containing the impedance gold electrodes in 60 µl medium and differentiated. Impedance was measured intermittently using the RTCA unit in a cell culture incubator. At time of exposure impedance was measured every 5 min for the 24-h duration. Cell index (CI) was normalised to the impedance before measurement per well.

### TempO-Seq assay

Three separate batches of HepaRG and RPTEC/TERT1 were cultured in separate medium stocks and seeded and differentiated in 12-well plates. The HepaRG and RPTEC/TERT1 experiments were conducted at the VUB and the MUI, respectively. The three biological replicates of differentiated cells were exposed to chemicals, medium controls and 0.1% DMSO controls for 24 h in serum-free medium. Morphology was documented by phase contrast microscopy (Fig. 2). Wells were washed in 1 ml PBS (DPBS, Gibco, 14190-094) and lysed in 750 µl of 1× BioSpyder lysis buffer. Lysates were frozen at − 80 °C and shipped to BioSpyder technologies on dry ice where the TempO-Seq assay was conducted.

Cellular extracts were harvested for TempO-Seq analysis to quantify the 3050 probe set representing 2839 genes (Table S1). A pair of detector oligos were annealed to adjacent 25 nt sequences in the target RNA, after which excess oligos were digested with a nuclease, and remaining oligos were ligated (Yeakley et al. 2017). This process occurred as a homogenous assay through progressive dilution to ensure enzyme compatibility and was, therefore, free of the need for bead-based clean-up or poly (A) + selection. Furthermore, the assay did not require RNA purification or cDNA synthesis. Once ligated, the detector oligos acted as templates in a PCR-based amplification to add sequencing adapters for an Illumina instrument and sample barcodes. Since all the detector oligos share the same pair of primer landing sites, the assay could be multiplexed with respect to the RNA targets, but still single plex with respect to the primers. The PCR primers both include a 9-mer index sequence, different for the primers used to amplify each sample, that resulted in dual index sample barcodes, allowing pooling post-PCR of up to 384 samples in 1 library. The number of samples per library depends on the sequencing depth achievable for a given instrument and the read depth desired. For a detector pool of ~ 3 K targets, between 0.5M and 3M reads per sample should give correlations of R^2^ = ~ 0.97 for technical replicates of 100 ng purified total RNA. Each sample fastq file was aligned against the TempO-Seq transcriptome using the Bowtie aligner (Li and Durbin 2009). The output of this analysis generated a table of counts per gene per sample.

### Lactate assay

Supernatant samples from the same TempO-Seq run were centrifuged at 150 g for 5 min to remove free floating cells. The supernatant was frozen at − 20 °C until assay. Samples from the VUB lab were transferred to the MUI lab for assay. In a 96 well microtitre plate 10 µl supernatant medium was incubated with 90 µl lactate reagent buffer (86 mM Triethanolamine HCl, 8.6 mM EDTA.Na_2_, 33 mM MgCl_2_, 326 µM N-methylphenazonium methyl sulphate (PMS), 790 µM p-iodonitrotetrazolium violet (INT), 3.37 mM β-NAD, 7% ethanol, 0.4% Triton-X-100, 4 U/ml Lactate Dehydrogenase) for approximately 7 min at room temperature, as previously described (Limonciel et al. 2011). Optical density was measured in a Tecan Infinite M200 at 490 nm. An 8-point calibration curve starting with 25 mM lactate was used to determine actual concentrations. To avoid confusion we wish to clarify that this assay measures the metabolite lactate, not the activity of the lactate dehydrogenase enzyme, which is often used as a cell viability (plasma membrane integrity) assay.

### Data analysis and visualisation

TempO-Seq sample outliers were assessed for the two cell types independently and identified using both the Tukey’s and Grubb’s outlier tests. No outliers were uncovered in this sample set. Normalization and differential expression were performed using the DESeq2 package (Love et al. 2014). To perform differential expression analysis, each treatment condition was paired with the appropriate control and the counts for each sample were then normalized using the DESeq2 estimateSizeFactors function. Differential expression of each treatment relative to its respective control was measured using the Wald test. Probes with Benjamin Hochberg adjusted *p* values ≤ 0.05 were considered significantly differentially expressed.

Heat maps were generated using the conditional formatting function of Microsoft Excel (version 1803). Genes were assigned to ATF4, Nrf2 or p53 pathways using information from several published sources (Riley et al. 2008; Limonciel et al. 2015). For a complete list of pathway allocations and sources see Table S3. Toxicological Prioritization Index (ToxPi) visualisations were generated using the National Center for Computational Toxicology, U.S. EPA’s ToxPi software version 2.0 (Reif et al. 2013; Marvel et al. 2018). The linear algorithm was used for all Pis, with equal weightings. The complete input file for the ToxPi software is given in Table S5.

### Statistical analysis

For concentration dependent analysis a one-way ANOVA with Dunnett’s post-test vs control (lactate and individual genes) was conducted. For time and concentration analysis a two-way ANOVA was used with a Sidak’s post-test (xCELLigence). Both were generated using GraphPad Prism version 6. See legends for specific details.

## Results

**Fig. 1:**
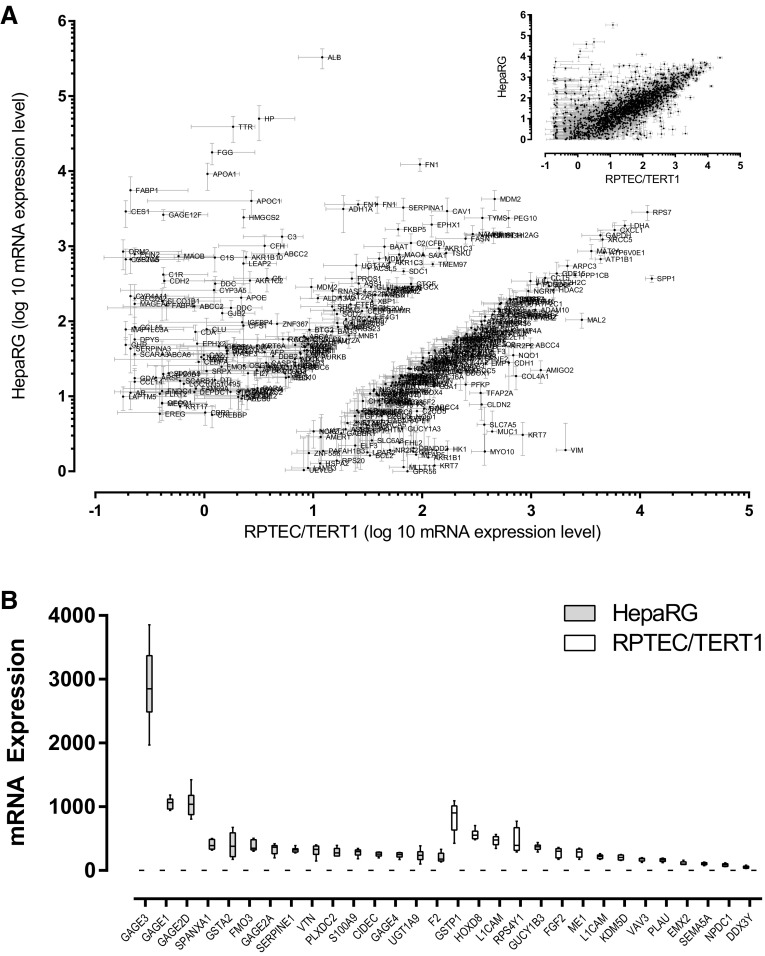
Comparison of HepaRG and RPTEC/TERT1 transcriptome. From a total of 3050 probes (inset **a**), probes were filtered for significant difference between the in vitro models (*p* value < 0.001) and with a coefficient of variance greater than 0.6. **a** Plot of HepaRG mRNA values against the corresponding RPTEC/TERT1 values. **b** Most abundant 15 genes from the 105 genes which are exclusively expressed in 1 model. All values are given in Table S2A and S2B

The control samples, consisting of three medium controls and three 0.1% DMSO treated controls per cell line, were used to compare the baseline gene expression of differentiated HepaRG and RPTEC/TERT1 cells (Fig. 1 and Table S2). While RPTEC/TERT1 and HepaRG exhibited a similar expression of the majority of genes (Fig. 1a inset, Table S2A), 519 genes had significantly different basal expression levels in the two cell lines (Fig. 1 main, Fig. 1b and Table S2B). HepaRG exhibited significantly higher expression of genes encoding plasma proteins, including albumin (ALB), haptoglobin (HP), transthyretin (TTR), alpha fetoprotein (AFP), alpha-2-macroglobulin (A2M), apolipoproteins (APOA1, APOC1, APOE), fibrinogen (FGG, FGB) and complement proteins (C3, C1R and CFH), genes involved in xenobiotic metabolism, including cytochrome P450s (CYP3A4, CYP3A5, CYP2E1), N-acetyltransferase 2 (NAT2), carboxylesterase 1 (CES1), and other known liver-specific genes, including fatty acid binding protein 1 (FABP1), orosomucoid 1 and 2 (ORM1, ORM2), ABCC2, alcohol dehydrogenase (ADH1A and 1B), SLCO1B1, SLCO2B1, bile acid-CoA:amino acid N-acyltransferase (BAAT), genes involved in the urea cycle Arginase 1 (ARG1) and the transcription factor hepatocyte nuclear factor 4 alpha (HNF4A). HepaRG, compared to RPTEC/TERT1 cells also highly express Epiregulin (EREG) and several genes associated with cancer, many belonging to the GAGE family (GAGE − 1, − 2, − 3, − 4 and − 12).

RPTEC/TERT1 cells exhibited a higher expression of genes including the nephrogenic transcription factor PAX8, the tight junction protein claudin 2 (CLDN2), SLC7A5 and SLC3A2 (the genes encoding the proteins for the large neutral amino acid transporter LAT1), the brush border enzyme gamma glutamyl transferase (GGT1), collagen 4A (COL4A), methionine adenosyltransferase 2A (MAT2A), secreted phosphoprotein 1 (SPP1), mal, T-cell differentiation protein 2 (MAL2), ATP binding cassette subfamily C members (ABCC 4 and 5), phosphofructokinase (PFKP), gamma-butyrobetaine hydroxylase 1 (BBOX1), vimentin (VIM), ATPase Na+/K+ transporting subunit beta 1 (ATP1B1), the proton pump (ATP6VOE1), glyceraldehyde 3-phosphate dehydrogenase (GAPDH), ribosomal protein S7 (RPS7), amyloid beta precursor protein (APP), myosin X (MYO10), NAD(P)H quinone dehydrogenase 1 (NQO1), mucin 1 (MUC1), adhesion molecule with Ig like domain 2 (AMIGO2) and plasminogen activator urokinase (PLAU).

**Fig. 2:**
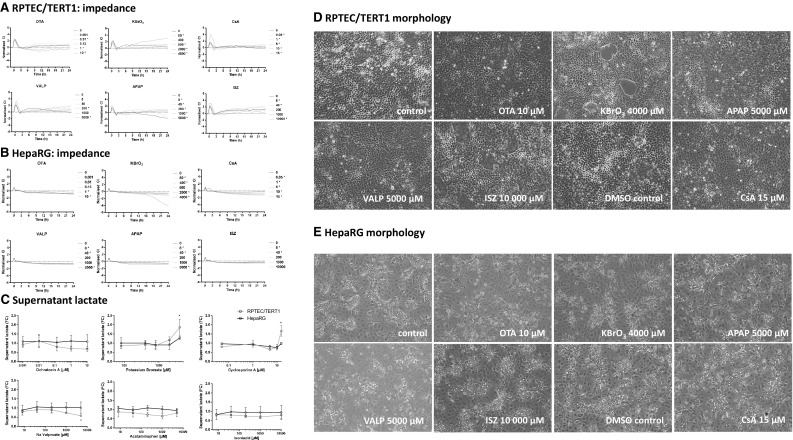
xCELLigence, lactate production and morphology after 24 h single application exposures. **a, b** Impedance measurements utilising the xCELLigence system with the same concentrations as utilised for the transcriptomics study. The values are mean from three biological replicates. *Indicates a significant difference using a two-way ANOVA, using a Sidak’s post-test with a significant cut off of 0.05. **c** Supernatant lactate from the same cultures utilised in the transcriptomic study. Values are mean fold control (FC) ± standard deviation. *Indicates a significant difference using a one-way ANOVA, using a Dunnett’s post-test with a significant cut off of 0.05. **d, e** Phase contrast morphology of cells with the highest concentration of compounds

Upon exposure to the selected compounds, temporal alterations in impedance utilising the xCELLigence system showed an initial spike after all treatments in both cell lines, presumably due to the manipulation of the cells outside the incubator (Fig. 2a, b). The xCELLigence apparatus measures net impedance of each well, reported as Cell Index (CI), with high temporal resolution (Kustermann et al. 2013; Kho et al. 2015). CI is dependent on the amount of cells attached to the well, the forces at which they bind and the net volume of the cells per well and is considered a sensitive label-free viability assay (Ke et al. 2011). RPTEC/TERT1 cells exhibited an oscillating CI, compared to the smooth CI of HepaRG cells. RPTEC/TERT1 monolayers due to vectorial solute and water transport, form raised areas called, domes (Wieser et al. 2008; Aschauer et al. 2013). Where a dome forms, the cells are no longer in contact with the electrode and thus do not contribute to impedance. Since, domes are dynamic and are formed and collapse overtime, the CI pattern appears ruffled. The strongest compound effect observed in RPTEC/TERT1 was with 4000 µM KBrO_3_. This condition resulted in an increase of CI, beginning at 9-h exposure (Fig. 2a), which is likely due to toxicity induced decrease in dome formation. In HepaRG 4000 µM KBrO_3_ also showed the strongest effect, with a decrease in CI starting at 15-h exposure (Fig. 2b). Enhanced glycolysis, as measured by supernatant lactate, was observed in RPTEC/TERT1 cells only, and only in two conditions: CsA 15 µM and KBrO_3_ 4000 µM (Fig. 2c). VALP caused a slight decrease in lactate production at the highest concentration in RPTEC/TERT1 cells (Fig. 2c). Phase contrast morphology shows a typical representation of differentiated RPTEC/TER1 cells and differentiated HepaRG cells; with a complete monolayer, cobble stone morphology and dome formation in the RPTEC/TERT1 controls (Fig. 2d) and a mixture of compact hepatocyte-like cells and biliary epithelial-like cells for HepaRG cells (Fig. 2e). Morphological aberrations were observed only with KBrO_3_ at 4000 µM in RPTEC/TERT1 cells, with small holes visible in the monolayer (Fig. 2d, e).

**Fig. 3:**
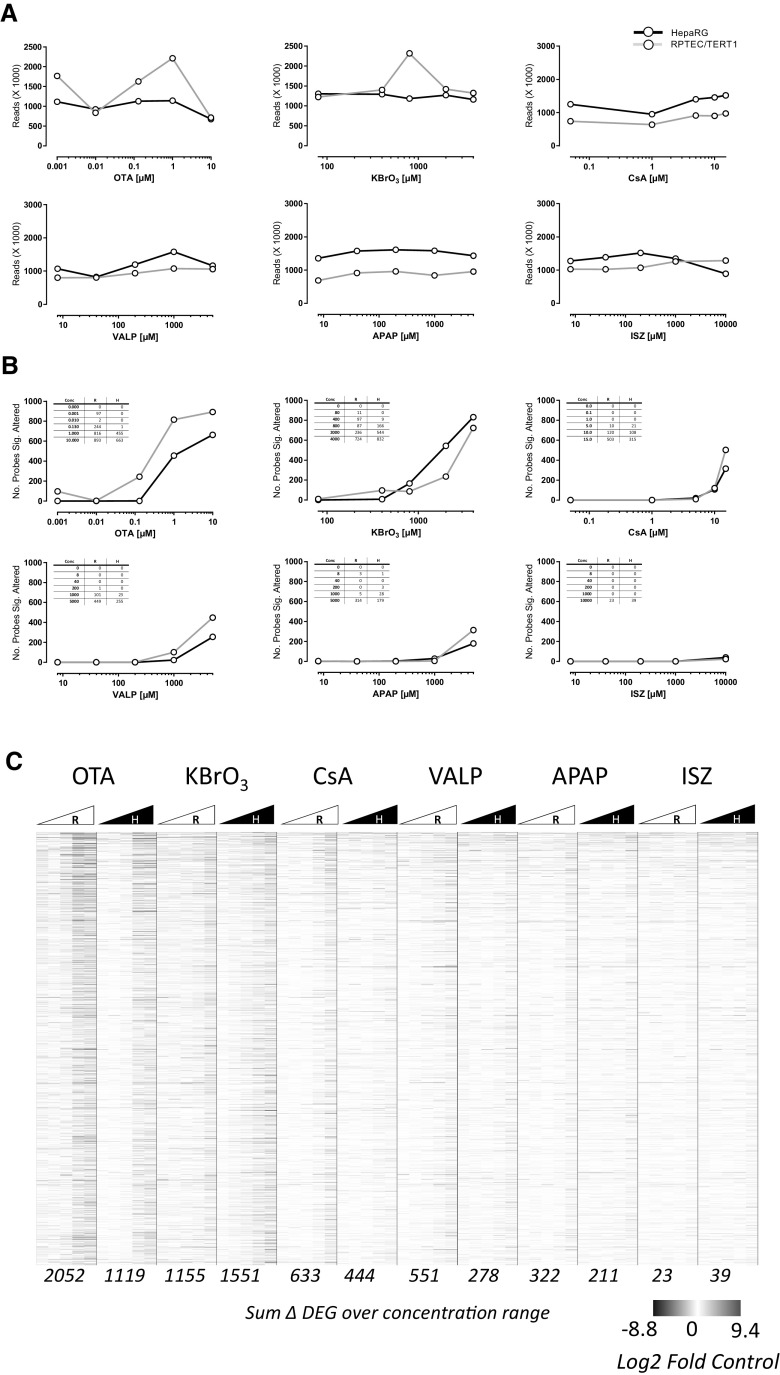
Effect of compounds on read counts (**a**), differential gene expression per treatment (**b**) and individual differential gene expression (**c**). The total read counts (×1000) are plotted versus concentration per cell model. **b** The number of total differentially expressed genes (with a significance cut off Benjamini Hochberg adjusted *p* > 0.05), are plotted versus concentration per cell model. The numeric values are given in the tables. **c** Heat map of log2 fold control data from 1514 genes that were significantly altered in 2 or more conditions. The heat map is sorted by the sum across all conditions per gene. R is RPTEC/TERT1, H is HepaRG. The red colour indicates increased and blue decreased, where white is unchanged. Where the log2 fold control could not be calculated (i.e., a division by zero error where all values zero in the control) the value is also represented by a white. The values at the bottom of the heat map are the sum of the DEGs over the 5 concentrations per cell line (maximum possible is 7570). The values behind the heat map with the gene annotations are given in Table S4

Figure 3 shows the read counts, differentially expressed probes (DEP) and a heat map of genes which were differentially expressed compared to controls in at least 2 of the 60 conditions (i.e., cell type, compounds, and concentration). The treatments had no discernible effect on read counts (Fig. 3a). There was, however, a concentration-dependent increase (i.e., an increase in at least two consecutive concentrations) in the number of DEPs for all compounds except ISZ. OTA had the strongest impact on DEPs, followed by KBrO_3_, CsA, VALP, APAP, and ISZ. (This does not reflect the potency ranking as different concentrations were used.) Based on the first concentration with an increase in DEP, RPTEC/TERT1 were more sensitive to OTA and KBrO_3_, and sensitivities for the rest of the compounds were similar.

**Fig. 4:**
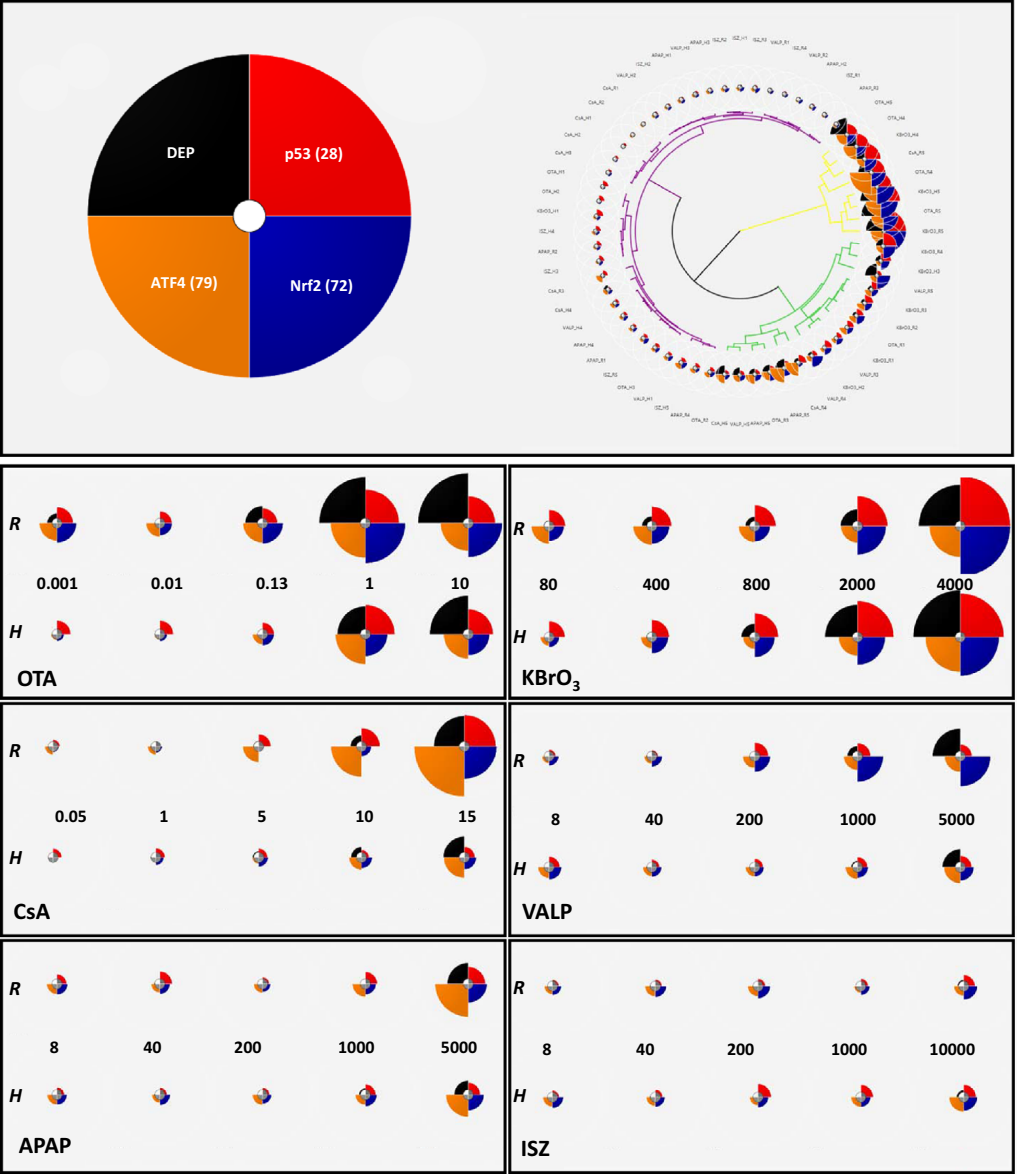
Representation of compound-induced differentially expressed genes using ToxPi visualisations. The ToxPi v 2 software was used to generate the diagrams. The top panel shows a full pie with its colour codes and gene numbers each slice uses the linear model. DEP is differentially expressed probes. The numbers for p53, Nrf2 and ATF4 represent the no. of genes allocated to those pathways (see Table S3). On the right of the top panel is a Hierarchical Clustering using ward.D2. Labelling of samples is compound code, the dilution (as per Table 1), R for RPTEC/TERT1 and H for HepaRG The lower panels show the ToxPis per cell type, compound and concentrations, where numbers represent the µM concentration of the corresponding compound. The ToxPi data are given in Table S5.

The data are also visualised using ToxPi with the slices representing DEP, genes in the ATF4 (major branch of the unfolded protein response), Nrf2 and p53 pathway (Fig. 4). It should be noted that the ToxPi charts do not show directionality of the genes in the pathway, just overall impact. Utilising the hierarchical clustering feature, three main clusters were apparent (Fig. 4). The yellow cluster represents the concentrations that had the highest impact on the four parameters and includes the two highest concentrations of OTA and KBrO_3_ for both cell types (OTA_H4,H5, OTA_R4,R5, KBrO_3__H4,H5, KBrO_3__R4,R5) and the highest concentration of CsA in RPTEC/TERT1 cells (CsA_R5). The green cluster represents the conditions that had a medium impact and the purple cluster contains mild to zero impact. All of the ISZ compounds were in the green cluster. In the lower panels of Fig. 4 the ToxPis are organised per compound and cell type. OTA had a strong impact on all three pathways, but the impact was larger in RPTEC/TERT1 cells. KBrO_3_ had a strong impact the Nrf2 and p53 pathways. Both CsA and APAP had a stronger impact on the ATF4 pathway and this was larger in RPTEC/TERT1 cells. In RPTEC/TERT1 cells VALP had a prominent effect only on the Nrf2 pathway. None of the selected pathways were impacted on by ISZ in either cell type.

**Fig. 5:**
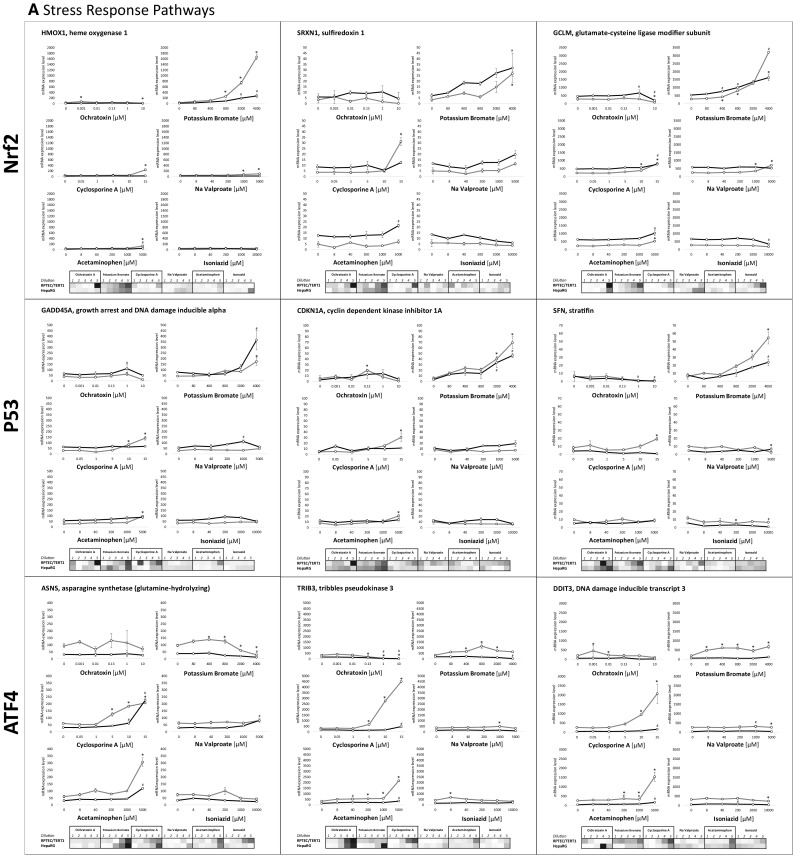

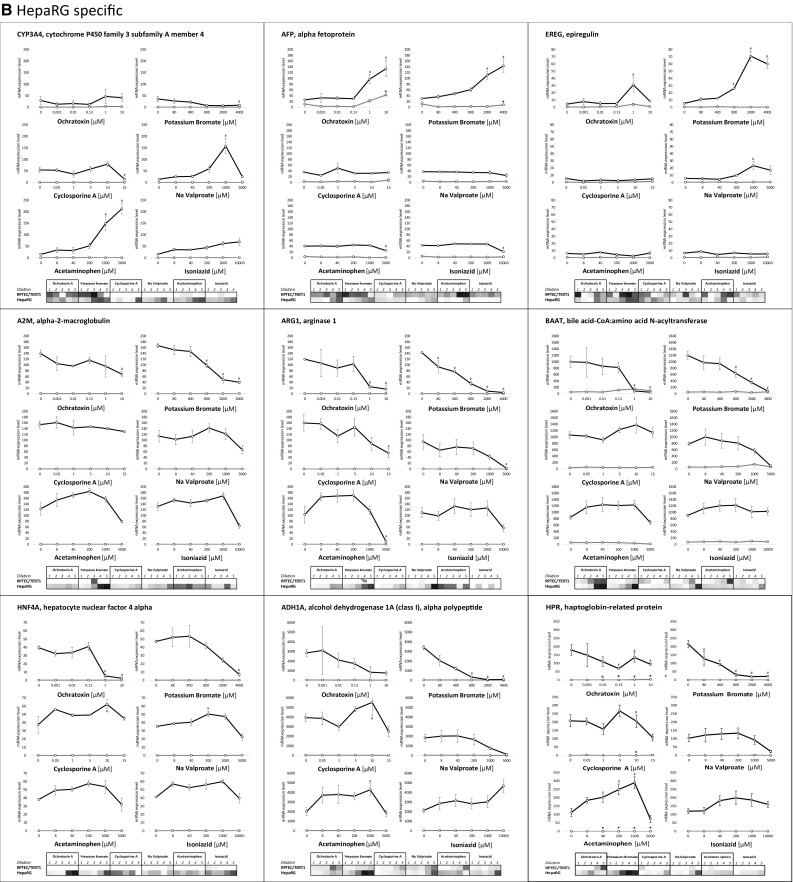

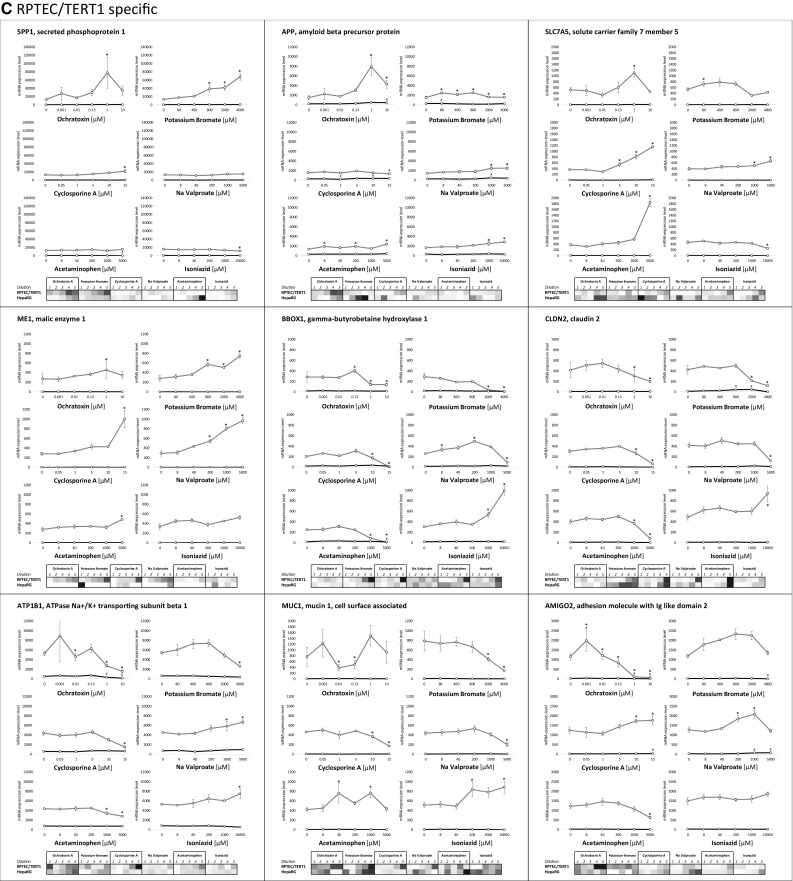
Concentration response relationships of selected genes, representing **a** stress response pathways, **b** HepaRG-specific and **c** RPTEC/TERT1-specific. In the graphs the values are expressed as mean mRNA expression ± SEM. In the heat map values represent the mean log2 fold control with red representing the highest value and blue the lowest. White represents either 0 or absent. Statistical significance to control (*p* < 0.05) is denoted by * for RPTEC/TERT1 and # for HepaRG using a one-way ANOVA, with a Dunnett’s post-test and significant cut off of 0.05

Quantitative data for 27 selected representative genes is given graphical form as non-transformed and in heat maps as log2 fold control transformed data (Fig. 5). The first panel of nine represent genes in the Nrf2 (HMOX1, SRXN1 and GCLM), p53 (GADD45A, CDKN1A, SFN) and ATF4 pathways (ASNS, TRIB3, DDIT3) (Fig. 5a). For the Nrf2 genes, HMOX1 was more responsive in RPTEC/TERT1 cells, while SRXN1 and GCLM showed similar levels of induction (Fig. 5a). KBrO_3_ was the strongest inducer of these genes. At the highest concentration of OTA HMOX1 was significantly decreased in RPTEC/TERT1 and GCLM significantly decreased in both cell types. For the p53 genes, GADD45A, CDKN1A and SFN showed similar levels of induction in both cell types and again KBrO_3_ was the highest inducer. OTA decreased SFN at the highest 2 concentrations in HepaRG cells. For the ATF4 genes, both TRIB3 and DDIT3 were more responsive in RPTEC/TERT1, whereas ASNS showed a similar level of maximum induction in both cell types. CsA, particularity in RPTEC/TERT1 cells, was the highest inducer of these three genes, but the highest concentration of APAP also resulted in a significant increase in ATF4 genes in both cell types. OTA decreased TRIB3 in both cell types.

Examples of HepaRG responsive genes are given in Fig. 5b. CYP3A4, EREG, A2M, ARG1, HNF4A, ADH1A were unresponsive in RPTEC/TERT1 cells. AFP, BAAT and HPR showed some mild induction in certain exposures in RPTEC/TERT1. All of these 9 genes were robustly responsive with at least one of the compounds in HepaRG. APAP was a strong inducer of CYP3A4; OTA and KBrO_3_ were inducers of AFP; while KBrO_3_ and VALP were inducers of EREG. A2M, ARG1, BAAT, HNF4A, ADH1A and HPR were mostly decreased in the chemical exposures, which was particularly predominant with KBrO_3_ exposure.

Examples of RPTEC/TERT1 responsive genes are given in Fig. 5c. SPP1, SLC7A5, ME1, BBOX1, and MUC1 were unresponsive in HepaRG cells. APP, CLDN2, ATP1B1 and AMIGO2 showed some mild responses in HepaRG. All of these 9 genes were robustly responsive with at least three of the compounds in RPTEC/TERT1. SPP1 was induced by OTA, KBrO_3_ and CsA. APP was induced by all compounds except CsA. SLC7A5, which is also an ATF4 responsive gene, was induced by CsA, APAP and VALP. ME1, which is an Nrf2 responsive gene was induced by KBrO_3_, CsA and VALP. BBOX1, CLDN2, ATP1B1 and MUC1 were frequently decreased in chemical exposures, however, all were induced by ISZ. AMIGO2 was induced by CsA and VALP (VALP was biphasic), whereas OTA and APAP attenuated AMIGO2.

## Discussion

The main aim of this study was to investigate the use of the TempO-Seq platform for application to in vitro toxicology. Comparison of the base-line expression of the 2 cell types uncovered a clear separation of 519 genes (Fig. 1 and Table S2B). HepaRG cells differentially expressed genes related to their liver origin including; plasma proteins, metabolising enzymes, and mediators of fatty acid metabolism. There was also high expression of GAGE isoforms and EREG, which likely relates to their cancer origin as compared to the non-cancerous RPTEC/TERT1 cells (Wieser et al. 2008). The RPTEC/TERT1 cell line, differentially expressed genes involved in renal phenotype including; transport, tight junction formation, and energy metabolism. Thus, the targeted gene panel, and the cell types utilised, captured renal and hepatic phenotypes. Challenging the cells with six compounds at six concentrations revealed a clear concentration–response relationship in the total number of differentially expressed probes for all compounds except ISZ. (Here concentration–response is defined as an increase in at least two consecutive concentrations and ISZ had an effect only at the highest concentration). Taking into consideration the concentrations applied, the potency ranking was OTA > CsA > KBrO_3_ > VALP > APAP > ISZ. RPTEC/TERT1 cells were more sensitive to OTA and KBrO_3_, while both cell lines were similar sensitive to the other compounds. In the cellular assays and from phase contrast morphology only KBrO_3_ and/or CsA showed a strong effect and only at the highest concentration applied, thus transcriptomic signatures were far more sensitive than these assays.

An advantage of TempO-Seq (and other sequencing-based techniques) is the possibility to use linear mRNA counts. While log2 control readouts are useful, especially for visualisation, it can also reduce data to sometimes biological meaningless information, especially where untreated values are close to zero. This phenomenon is well represented here in the comparison of the non-transformed and the log2 transformed data of the HepaRG-specific and RPTEC/TERT1-specific compound responses (Fig. 5b and c, graphs vs heat maps).

Several mechanistic aspects of compound-induced effects could be garnered from the TempO-Seq analysis. OTA, a nephrotoxic carcinogenic mycotoxin in rodents (IARC 1993; Zhang et al. 2016), was the most potent compound used and exhibited a high impact on the selected pathways, i.e., ATF4, Nrf2 and p53 (Figs. 3, 4). However, this impact was uniquely an OTA-induced pathway suppression as none of the nine panel pathway genes were increased with increasing OTA exposures, in fact the majority were decreased by OTA (Fig. 5a). Similar observations have been reported with OTA previously in the context of Nrf2 suppression (Cavin et al. 2006; Boesch-Saadatmandi et al. 2008; Limonciel and Jennings 2013). OTA-induced suppression of stress responses may contribute, or even underlie, its toxicity and carcinogenicity. KBrO_3,_ a group 2B human genotoxic carcinogen, mediated by oxidant activity (DeAngelo et al. 1998; Limonciel et al. 2012), resulted in a strong concentration-dependent activation of the Nrf2 and p53 pathways in both cell types, while ATF4 was to a lesser extent activated. ASNS expression was even attenuated by KBrO_3_. Cyclosporine A (CsA) is a cyclophilin binding calcineurin inhibitor and potent immunosuppressant, but is also a competitive inhibitor of the bile salt export pump (ABCB11) (Starokozhko et al. 2017). CsA at supra-therapeutic concentrations (15 µM) has been shown to injure mitochondria and cause endoplasmic reticulum (ER) disturbances (Wilmes et al. 2013). Here, CsA showed predominately a strong activation of the ATF4 genes, which is in keeping with previous findings (Wilmes et al. 2013). The anti-epileptic compound sodium valproate (VALP) has been linked with liver steatosis and renal proximal tubular dysfunction, potentially via β-oxidation inhibition and mitochondrial dysfunction (Chang and Abbott 2006; Komulainen et al. 2015; Willebrords et al. 2015; Heidari et al. 2017). Here, VALP resulted in an activation of the Nrf2 pathway in both cell types and induced the ATF4 regulated gene ASNS in HepaRG cells. Acetaminophen (APAP), the widely used analgesic is well documented to cause liver injury in high doses due to formation of the metabolite N-acetyl-p-benzoquinoneimine (NAPQI) and subsequent NAPQI covalent thiol reactivity, glutathione depletion, resulting in proteotoxicity and ER stress (Bessems and Vermeulen 2001). Here, APAP had a prominent effect on ATF4 genes in both cell types. The major target organ of INZ is considered to be the liver, where INZ metabolites are thought to bind covalently to proteins (Hassan et al. 2015; Metushi et al. 2016; Iorga et al. 2017). However, ISZ had the lowest impact here, and we did not observe an impact on any of the pathways investigated.

Many of the cell type-specific genes were also altered in response to compound exposures; 18 of these were selected for deeper analysis, 9 for HepaRG and 9 for RPTEC/TERT1. The hepatic phase I metabolising gene, CYP3A4 was significantly induced in HepaRG by APAP and VALP, the latter having a biphasic response. Both compounds have been previously shown to be CYP3A4 inducers (Feierman et al. 2002; Cerveny et al. 2007). CsA and KBrO_3_ decreased CYP3A4 expression. CsA suppression of CYP3A4 has also been previously reported (Amundsen et al. 2012). AFP was strongly induced by the carcinogens OTA and KBrO_3_ in HepaRG cells and to a lesser extent in RPTEC/TERT1 cells. AFP is a well-recognised marker of a hepatic foetal phenotype and is also discussed as a marker of hepatocellular carcinoma (Terentiev and Moldogazieva 2013). EREG, also a cancer associated protein (Riese and Cullum 2014), was induced by OTA, KBrO_3_ and VALP only in HepaRG cells. A2M, ARG1, BAAT, HNFA4, ADH1A and HPR were also affected by compound exposure and were for the most part down regulated. These genes are predominately liver expressed and HNF4A, for example, has been shown to be important in the maintenance of hepatic differentiation, where its overexpression can rescue dedifferentiation processes (Späth and Weiss 1997). Globally the described alterations of these nine genes could be interpreted as compound-induced dedifferentiation of the hepatic phenotype.

SPP1 was induced by OTA, KBrO_3_ and CsA in RPTEC/TERT1 cells. The induction of SPP1, which is more predominately expressed in native kidney tissue, has been previously associated with renal and not hepatic injury in rats in vivo (Dadarkar et al. 2010). APP, which has role in neural growth and repair (Dawkins and Small 2014) was induced by all compounds, with the exception of CsA. SLC7A5, is involved in large neutral amino acid reabsorption, is strongly expressed in renal tubules and is also inducible by ATF4 (Han et al. 2013). SLC7A5 was induced by APAP, CsA and VALP. ME1, which is mildly expressed in the kidney in vivo under basal conditions, but is also under the transcriptional regulation of Nrf2 (Wu et al. 2011) was heavily increased by KBrO_3_, CsA and VALP in RPTEC/TERT1 cells. BBOX1, CLDN2, ATP1B1, MUC1 and AMIGO2 were for the most part decreased by the compound panel. Since these five markers are highly expressed in kidney cells, their decrease may reflect a compound-induced dedifferentiation of the proximal tubule phenotype. ISZ, however, induced BBOX1, CLDN2, ATP1B1 and MUC1, whereas VALP induced ATP1B1, indicating a somewhat more complicated interpretation for certain compounds.

In summary, RPTEC/TERT1 and HepaRG have similar sensitivities to the chosen compounds and exhibit both common and cell type-specific responses. KBrO_3_ as expected had a clear activation of the Nrf2 and p53 pathways. Whereas, CsA and APAP were strong inducers of the ATF4 pathway. AFP and EREG, were often upregulated upon exposure in HepaRG cells, while A2M, ARG1, BAAT, HNF4A, ADH1A and HPR were most often down regulated. In RPTEC/TERT1 cells, SPP1, APP, SLC7A5 and ME1 were commonly induced, while BBOX-1, CLDN2, ATP1B1, MUC1 and AMIGO2 were often decreased. SLC7A5 and ME1 appear to be renal-specific pathway reporters of ATF4 and Nrf2, respectively.

In conclusion, this study shows that TempO-Seq is a robust and sensitive method to quantify chemical-induced transcriptomic alterations and also highlights the usefulness of quantitative transcriptomics to identify mechanistic and tissues-specific effects of chemicals.

## Electronic supplementary material

Below is the link to the electronic supplementary material.

Supplementary material 1 (XLSX 2835 KB)
